# Cardiomyopathy: Evaluating Disparities in Place of Death in the United States Using the CDC Wide-Ranging Online Data for Epidemiologic Research (CDC WONDER) Database Over 22 Years

**DOI:** 10.7759/cureus.46645

**Published:** 2023-10-07

**Authors:** Hussein Al Hussein, Dhruvkumar N Jadav, Aruna Anantharaj, Shan Doghouz, Nisha S Kolhe, Jyoti Thapa, Hamza Asif

**Affiliations:** 1 Internal Medicine, Hamad Medical Corporation, Doha, QAT; 2 Medicine, Pandit Deendayal Upadhyay (PDU) Medical College, Rajkot, IND; 3 Internal Medicine, Wuhan University School of Medicine, Hubei, CHN; 4 Internal Medicine, Charles University, First Faculty of Medicine, Prague, CZE; 5 Internal Medicine, Anna Medical College and Research Centre, Montaigne Blanche, MUS; 6 Internal Medicine, Shandong University School of Medicine, Jinan, CHN; 7 Pulmonology, Khyber Teaching Hospital, Peshawar, PAK

**Keywords:** end-of-life care, demographic factors, disparities, place of death, cardiomyopathy

## Abstract

Background

The human experience involves the inevitable end of life, whether sudden or expected. Ensuring a dignified end-of-life encounter necessitates understanding influential factors. Cardiomyopathy, a group of heart muscle diseases, has varying mortality implications, including heart failure and arrhythmias. Disparities in place of death (hospital, home, or hospice) can significantly alter the end-of-life care for a patient.

Methods

The aim of this study is to identify variations in death locations for U.S. cardiomyopathy patients between 1999 and 2020, based on age, gender, race, and census region, utilizing the CDC WONDER ( CDC Wide-Ranging Online Data for Epidemiologic Research) database, which contains a wide array of public health information. Data were categorized by age, gender, race, and location, and further subcategorized according to place of death. Statistical analysis was done via R programming software.

Result

The aggregate data of 528,401 cardiomyopathy-related deaths from 1990 to 2020 were obtained. Findings revealed age, gender, and regional disparities in death location. Notably, cardiomyopathy is found to be prevalent in the 75+ years age group, male gender, and people belonging to Caucasian descent, and maximal in the Southern census area. The study’s logistic regression analysis unveiled a significant association between demographic factors and death locations.

Conclusion

This research underscores the significance of understanding disparities in the place of death for cardiomyopathy patients, shedding light on demographic influences and paving the way for patient-centered end-of-life care approaches.

## Introduction

The inevitable end of life, whether anticipated or sudden, is a vital part of the human experience [1]. As we strive to provide patients with a dignified end-of-life experience, it becomes crucial to understand the variables that can significantly impact this experience [2,3]. One such variable is the location of death, such as hospitals, medical facilities, nursing homes, hospices, or at home. Hospitals and nursing centers often provide superior end-of-life care, offering relief from symptoms through palliative care [4]. However, home and hospice care may not always offer the same level of efficiency, potentially leading to painful deaths. Despite advancements in healthcare, disparities in the place of death remain.

Another crucial factor impacting end-of-life experiences is the specific health condition a person is dealing with. Among these conditions, heart diseases, including various forms of cardiomyopathy, represent a significant proportion. Cardiomyopathy is a group of diseases that affect the heart muscle. It includes prevalent forms like hypertrophic and dilated cardiomyopathies, along with rarer variants such as arrhythmogenic right ventricular, restrictive, Takotsubo, and left ventricular non-compaction cardiomyopathies [5]. Understanding the patterns and disparities in places of death is essential to inform physicians and patients, aiming to foster end-of-life care that aligns with the patients' wishes and values.

The aim of this study is to determine variations in place of death such as medical facilities, nursing homes, residential homes, hospice, or other locations for patients with cardiomyopathy in the United States from 1999 to 2020, based on age, gender, race, and census region using the CDC WONDER (CDC Wide-Ranging Online Data for Epidemiologic Research) database.

## Materials and methods

This is a web-based cross-sectional observational study conducted in July 2023. The CDC WONDER database was used to collect relevant data for this study. This online database houses a wide variety of public health information freely accessible to the public [6].

All data were collected on July 11, 2023, from the CDC WONDER Underlying Cause of Death database. The database was queried for people of all races who died from cardiomyopathy between the years 1999 and 2020, inclusive: "1999-2020: Underlying Cause of Death by Bridged-Race Categories". The selection of the cause of death was based on the ICD-10 code for cardiomyopathy: I42 (Cardiomyopathy).

Data were categorized by four criteria: age, gender, race, and location. People of all ages were included and grouped into 10-year age brackets. All genders (male, female), races (American Indian or Alaska Native, Asian or Pacific Islander, Black or African American, White), and origins (Hispanic or Latino, Not Hispanic or Latino, Not Stated) were also included. Location was determined based on U.S. Census Regions, and all four regions were included (Northeast, Midwest, South, and West).

Data were further organized based on the place of death. All locations of death present in the database were sorted into three categories for this study: Home or Hospice (Decedent’s home, Hospice facility), Medical Facility or Nursing (Inpatient, Outpatient or ER, Dead on Arrival, Status unknown, Nursing home/long-term care), and Others (Other, Place of death unknown).

The data were exported to a Microsoft Excel sheet (Microsoft Corporation, Redmond, Washington, United States), and the total number of deaths based on the four criteria was summarized according to the place of death. Statistical analysis was performed using R programming software (R Core Team, Vienna, Austria). Univariate logistic regression was also used to analyze deaths at Home or Hospice, and odds ratios were calculated.

## Results

The aggregate data of 528,401 deaths from 1999 to 2020 were obtained for cardiomyopathy from the CDC WONDER database. Table 1 presents the total cardiomyopathy deaths categorized by places of death namely home/hospice, medical care/nursing facility, and others. Among those who received home/hospice care, the 85+ year age group reported the highest number of deaths, whereas the 1-4 year age group had the lowest number of deaths. For medical facility or nursing care, the 75-84 year age group accounted for the highest number of deaths, with the 1-4 year age group having the lowest. In the "Others" category, the highest number of deaths occurred in the 85+ year age group, while there were zero deaths reported in the 1-4 year age group.

**Table 1:** The total cardiomyopathy deaths categorized by places of death namely home/hospice, medical care/nursing facility, and others.

	Home or Hospice (n = 160,276)	Medical Facility or Nursing (n = 342,497)	Others (n = 25,628)	
Ten-Year Age Groups				
<1 year	134	2079	20	
1-4 years	68	687	0	
5-14 years	155	1045	18	
15-24 years	1142	3805	370	
25-34 years	3563	7704	965	
35-44 years	8703	15,810	2000	
45-54 years	17,060	29,735	3264	
55-64 years	23,042	45,070	3242	
65-74 years	27,511	62,628	3189	
75-84 years	38,028	88,179	4991	
85+ years	40,849	85,733	7537	
Gender				
Female	61,929	143,314	9905	
Male	98,347	199,183	15,723	
Census Region				
Census Region 1: Northeast	27,469	64,050	2727	
Census Region 2: Midwest	35,596	83,106	5056	
Census Region 3: South	60,315	133,254	11,778	
Census Region 4: West	36,896	62,087	6067	

Regarding gender differences, the number of deaths was higher for males across all locations. Additionally, in Census regions, the number of deaths reported in the South surpassed other regions in all categories.

Table 2 presents the predictors of home or hospice deaths. The univariate logistic regression revealed that individuals in the 45-54 year age group were more likely to die at home or hospice, taking the less than 1-year age group as the reference. Moreover, females had lower chances of death at home or in hospice, and when comparing Census regions, the Midwest showed the lowest likelihood of death in hospice care, using the West as the reference.

**Table 2:** The predictors of home or hospice deaths.

Variables	Univariate Logistic Regression		
	Odds Ratio	95% Confidence Interval	P-value
Age			
< 1 year	1.000 (Reference)		
1-4 years	1.550	(1.144, 2.102)	.005
5-14 years	2.284	(1.792, 2.911)	< .001>
15-24 years	4.285	(3.556, 5.163)	< .001>
25-34 years	6.438	(5.383, 7.7)	< .001>
35-44 years	7.654	(6.416, 9.132)	< .001>
45-54 years	8.098	(6.794, 9.653)	< .001>
55-64 years	7.471	(6.269, 8.903)	< .001>
65-74 years	6.548	(5.495, 7.801)	< .001>
75-84 years	6.393	(5.367, 7.616)	< .001>
85+ years	6.860	(5.759, 8.173)	< .001>
Gender			
Male	1.000 (Reference)		
Female	.883	(0.873, 0.894)	< .001>
Census Region			
Census Region 1: Northeast	.760	(0.746, 0.774)	< .001>
Census Region 2: Midwest	.746	(0.733, 0.759)	< .001>
Census Region 3: South	.768	(0.756, 0.78)	< .001>
Census Region 4: West	1.000 (Reference)		

**Figure 1:**
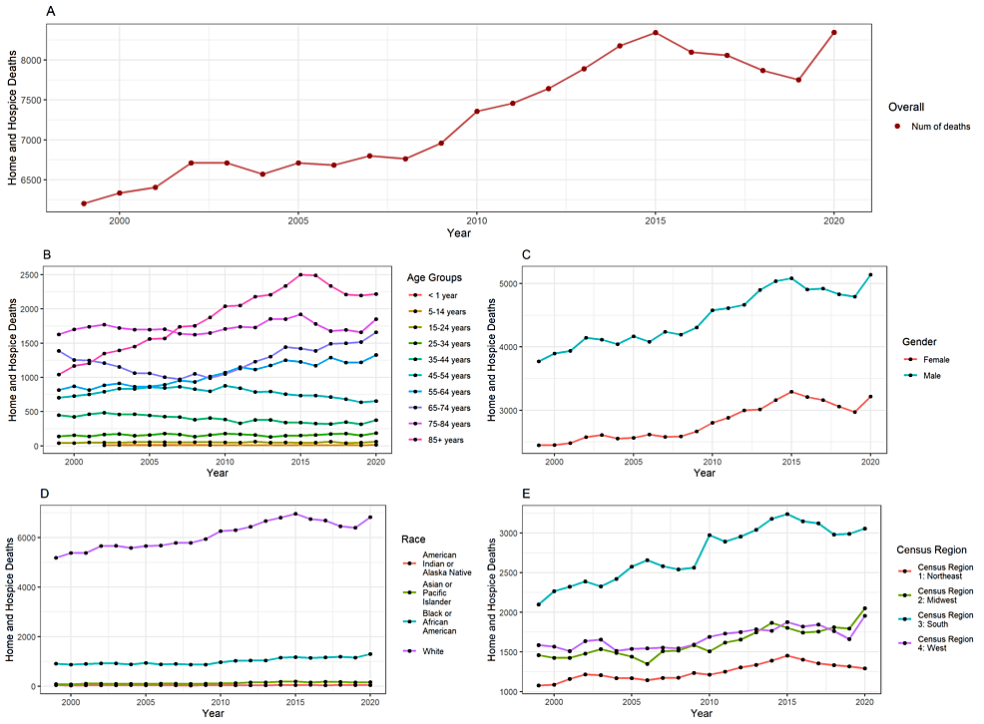
Home or Hospice death trends.

Figure 1a illustrates the overall number of Home/hospice deaths from 1999 to 2020, exhibiting an increasing trend year by year. While the number of deaths remained relatively stable from 2003 to 2008, a clear upward trend emerged after that, with a slight decrease in the trend reported for 2018-2019. Figure 1b provides an age group-wise analysis of home or hospice deaths. The 85+ age group accounted for the highest number of deaths, while the 5-14 year age group reported the lowest. An increasing trend year by year was evident in the 85+ year age group, while all other age groups showed relatively stable death trends. Figure 1c reveals a disparity in deaths between genders, with a higher number of deaths reported in males. A slight increasing trend year by year was observed for both males and females. Figure 1d displays the distribution of deaths across different races. The White race reported the highest number of deaths, whereas American Indian/Alaskan Natives had the lowest number of deaths. Figure 1e analyzes deaths in different Census regions, with the South showing the highest number of deaths and the Northeast reporting the lowest. An increasing trend in deaths is evident for the South Census region.

## Discussion

In our study investigating variations in place of death for patients with cardiomyopathy in the United States, the extensive timeframe allowed us to discern compelling patterns based on age, gender, race, and census regions.

Dying in the place of the patient's choice is viewed as a crucial element of end-of-life care, with the majority of patients expressing a preference to pass away at home regardless of their underlying health condition [7]. Home and hospice care saw the highest number of deaths in the 85+ year age group. Dying in such settings offers numerous potential benefits, such as reducing medical interventions, creating a comfortable space for spending time with loved ones, and granting the patient greater control over their surroundings [8]. In contrast, for Medical Facility or Nursing care, the 75- to 84-year age group accounted for the highest number of deaths, suggesting demand for intensive medical interventions among this age group. While all other age groups exhibited relatively stable death trends, an increasing trend year by year was evident in the 85+ year age group opting for home/hospice care. This finding was consistent with another report, as mortality rates from heart failure trended up since 2012 starting in elderly ≥75 years old following a period of steady decline [9]. The reasons behind these age-related variations warrant further investigation, as they may be influenced by factors such as the availability of home-based care services, familial support, medical complexities, and individual preferences.

Furthermore, based on univariate analysis, it was found that patients in the 45- to 54-year age group had a higher likelihood of passing away at home or in hospice care when compared to other age groups. Interestingly, this study aligns with other research indicating an increase in cardiovascular mortality among young adults [10,11]. The increased mortality within this particular age group signifies a more severe underlying illness and a less favorable prognosis. Consequently, this heightened risk may contribute to a higher inclination toward opting for home or hospice care. Understanding the unique preferences and concerns of patients can guide healthcare practitioners in delivering patient-centered care that aligns with their values and preferences for end-of-life care. Additionally, targeted interventions and supportive resources can be tailored to meet the needs of patients in this age range, optimizing the quality of their end-of-life experience.

Our study found a higher number of deaths among males compared to females. Remarkably, females had lower probabilities of dying at home or receiving hospice care throughout the entire study period. Women face a disadvantage towards the end of life due to outliving their spouses, which leads to a reduction in available caregivers and resources to support them [12]. Understanding the factors contributing to this disparity can inform interventions to bridge the gap and ensure that females have equal access to home and hospice care options. Moreover, addressing gender-related disparities in end-of-life care choices is crucial for promoting equitable and patient-centered care for patients with cardiomyopathy. By considering gender-specific needs and preferences, healthcare professionals and policymakers can strive to ensure that all patients with cardiomyopathy receive compassionate and patient-centered end-of-life care, regardless of their gender.

We also report significant racial and ethnic disparities, with white patients having the highest number of home or hospice deaths, while American Indian/Alaskan Natives reported the lowest. These results align with another report that also demonstrated a higher likelihood of cardiovascular mortality occurring in a hospice facility or at home for white individuals, compared to their black counterparts [13]. These racial disparities may be the result of underlying treatment bias as well as unfavorable socioeconomic health factors [14].

Significant differences in mortality across various geographical regions in the United States were also noted, with the South reporting the highest number of deaths in all categories. An increasing trend in home and hospice deaths was also evident for the South Census region over the 22-year study period, while the Northeast reported the lowest number of deaths. This aligns with past research showing regional disparities in heart failure and stroke mortality, where higher rates are concentrated in Southern states, earning the region the moniker "stroke belt" [15,16]. Similarly, data indicates that the prevalence of comorbidities like diabetes, hypertension, and obesity, as well as behavioral risk factors, shows a specific pattern across different geographical regions [16]. Unfortunately, unfavorable trends in risk factors including diabetes and obesity could result in increased rates of cardiovascular disease mortality, as is already the case [14,17]. Enhancing public health awareness regarding these harmful health behaviors may contribute to reducing the regional discrepancy in cardiomyopathy-related mortality.

Limitations

One of the limitations of our study is that the latest data from 2021 to 2023 is not included in the study due to its unavailability on the CDC WONDER database. Secondly, there are many different subcategories of cardiomyopathy, including dilated, hypertrophic, and restrictive cardiomyopathy but we did not further categorize our findings in order to keep things easier and simpler and to maintain a homogeneous group. Future studies can be done based on each subcategory when the data are available.

## Conclusions

We conclude that there is an overall rising trend in the rate of home and hospice deaths over the years in the United States with a dip seen between 2015 and 2019 that might be attributed to hospitalization and subsequent hospital deaths related to the coronavirus pandemic. For patients with cardiomyopathy-related deaths in the United States, age, gender, race, and census region are important considerations while interpreting the mortality trends. The trend is found to be more in the over 75 years age group, male gender, people belonging to Caucasian descent, and maximal in the Southern census area. Future studies should be directed to find the cause of increasing home and hospice deaths in the above-mentioned groups and how to provide them with better healthcare facilities.
